# Women leaders perceived barriers and consequences of safe abortion in Rwanda: a qualitative study

**DOI:** 10.1186/s12905-023-02366-4

**Published:** 2023-04-28

**Authors:** Lawrence Rugema, Marie Ange Uwase, Athanase Rukundo, Vianney Nizeyimana, Theobald Mporanyi, Aflodis Kagaba

**Affiliations:** 1 Health Development Initiative, PO Box 3955, Kigali, Rwanda; 2 School of Public Health, University of Rwanda, Kigali, Rwanda

**Keywords:** Women leaders, Unsafe abortion, Safe abortion, Adolescent sexual and reproductive health (SRH), Qualitative research, Content analysis, Rwanda

## Abstract

**Background:**

Between 2010 and 2014, approximately 25 million unsafe abortions were performed annually across the globe. Africa alone accounted for 29% of all unsafe abortions, and 62% of the related deaths. Women living in poverty, especially adolescents, lack information about where and how to access safe abortion services. They often lack adequate insight to make informed decisions. The purpose of this study was to explore the empowered perspectives of women leaders in Rwanda about the recent policy change for safe abortion. The study identifies women leaders’ perceived barriers and their attitudes about resulting consequences toward safe abortion.

**Method:**

In this qualitative study, seven focus group discussions and eight key informant interviews were performed in October 2019. A total of 51 women leaders participated, their age ranging from 38 to 60 years. Participants were drawn from three districts, namely Gasabo, Kicukiro, and Nyarugenge. For variability of data, participants came from parliament, government ministries, government parastatals, and civil society organizations. All interviews were conducted in Kinyarwanda and later translated into English. Data were analyzed using qualitative content analysis.

**Results:**

The emerging theme *Strong barriers and numerous consequences of safe abortion* illustrates how women leaders perceive barriers to safe abortion and its related consequences in Rwanda. The theme is divided into two categories: (1) *Perceived barriers of safe abortion* and (2) *Consequences of providing safe abortion*. The sub-categories for the first category are *Reluctance to fully support safe abortion due to perceived unjustified abortions”*, *Abortion-related stigma*, *Abortion is against cultural and religious beliefs*, *Emotional attachment to the unborn and Lack of awareness of abortion*. The sub-categories for the second category are *Perceived physiological trauma*, C*ause for barrenness/infertility*, I*ncrease in services abuse by adolescents/women*, *Increase of workload for healthcare providers*, *“Increase in sexual activities and STIs*, and *Abortion-related physiological trauma*.

**Conclusion:**

The subject of safe abortion evokes mixed reactions among participants, and is entangled with unsafe abortion in most cases. Participants stress that the word ‘abortion’ disturbs, regardless of whether it relates to being safe or unsafe. Participants believe the word ‘abortion’ outweighs the word ‘safe’. Societal expectations play a major role in the decision-making process of any adolescent or a family member faced with a pregnant adolescent regardless of the existing safe abortion law. Community mobilization and sensitization are crucial if safe abortion in accordance with abortion law is to be embraced. Messages that reinforce safe abortion as acceptable and address stigma, fears of trauma, and barrenness should be developed to educate adolescents, parents, and women leaders about safe abortion, to mitigate unsafe abortion-related complications.

## Background

Between 2010 and 2014, approximately 25 million unsafe abortions were performed annually across the globe, with the majority occurring in developing countries [1]. Africa accounted for 29% of all unsafe abortions, and 62% of unsafe abortion-related deaths [1]. It is estimated that 7 million women a year are admitted to hospital as a consequence of unsafe abortion performed in developing nations [2]. Moreover, the risk of dying due to induced unsafe abortion is believed to be the highest in Africa. Unsafe abortion is the second leading cause of death among women of reproductive age in Ghana [3]. The annual cost of treating unsafe abortion-related complications is approximately US$553 million [4]. According to the World Health Organization (WHO), unsafe abortion relates to terminating a pregnancy by persons who do not have the required technical knowledge in an environment that lacks either adequate or even basic medical standards [5].

The barriers to accessing safe abortion are multidisciplinary and vary at different levels of the community. Adolescents with unwanted pregnancies often resort to unsafe abortion due to such barriers as restrictive laws, poor services or a lack of services, high cost, abortion-related stigma, healthcare provider objections, mandatory waiting periods, mandatory counselling, misleading information, unnecessary medical tests that delay timely care, and the requirement for third-party authorization [6]. Pregnancy makes teenagers vulnerable to infection and increases their exposure to the high risks of abortion and obstetric complications [7]. Rwanda has ratified the Maputo Protocol (Protocol to the African Charter on Human and Peoples’ Rights on the Rights of Women in Africa) and allows access to safe legal abortion under five circumstances in its reformed 2019 penal code and changes as per the Ministerial Order N°002/MoH/2019. This was timely to increase access to safe abortion because there is, evidence to suggest that every year 24,000 women need emergency treatment for medical complications resulting from unsafe abortion [8]. At least 30% of these women did not receive any treatment for such complications due to fear of arrest in 2010 [8]. An estimated 16,749 women were treated for complications due to induced abortion in 2009 [9].

Thus, an unmet need for family planning and sexual and reproductive health and rights (SRHR) still existed by 2012, despite progress in the overall contraceptive coverage. A study conducted in the Southern Province of Rwanda among community health workers (CHWs) and nurses indicates that women’s reproductive decision-making is influenced by four factors: the cultural and historical precedent for large families; social pressure to conform to a husband’s beliefs; the assumption among some men that family planning is a woman’s issue; and barriers to quality service provision characterized by stock-outs, limited time with providers, and a prominent fear of side effects [10]. Several factors influence abortion among adolescents; in many countries, abortion is associated with a high socioeconomic status of women, educational attainment, and urban residence [11]. Rwanda made significant progress in sexual and reproductive health (SRH) between 2005 and 2015, as well as maternal and child health [12]. This has partly been attributed to the availability of a community-based health insurance scheme that improved access to health services and family planning [13].

According to the Rwanda Demographic and Health Surveys (RDHSs) [14, 15], contraceptive use among adolescents has improved over the years: from 3% of the adolescent population accessing contraception in 2005, to 24% in 2008, to 33% in 2010, and to 35% in 2015. The RDHSs indicate that an unmet need for family planning among adolescents trended down from 22% to 2005 to 4% in 2015 [15]. Yet teenage pregnancies increased, from occurring in 4.1% of the adolescent population in 2005, to 6.1% in 2010, and to 7.3% in 2018 [16]. Between 2009 and 2010 approximately 60,000 induced abortions were conducted in Rwanda [9]. A 2017 study conducted by the Health Development Initiative (HDI) to explore the causes, practices, and consequences of terminating a pregnancy found that an estimated 24% of women prisoners in Rwanda were incarcerated on abortion-related charges [17].

## Context of the study

### Abortion law

Rwanda revised its formerly strict abortion law in 2012. This change was attributed to advocacy initiatives by the Rwandan Youth Action Movement, a pressure group that highlighted the consequences young women face, including imprisonment, for seeking abortion [18]. The 2012 law describes the circumstances under which a pregnancy can be terminated. They include if the person requesting the abortion is a child, or became pregnant as a result of rape, forced marriage, or incest. In these circumstances, the law required the complainant to produce evidence in court for the grounds on which she is seeking an abortion before the court can grant permission for the procedure [19]. In 2019 a Ministry of Health ministerial order made changes to the abortion law, removing the requirement to go to court in order to seek an abortion [20]. The Ministerial Order N°002/MoH/2019 states that, “without prejudice to the provisions of Article 11 of this Order, the person requesting for abortion is not required to produce evidence of the grounds she invokes”. The order further states that if it is proved after abortion that the person who sought it provided false information, she is liable in accordance with the law [20]. Further, an abortion cannot be performed beyond 22 weeks’ gestation unless the mother’s life or the fetus is at risk [20].

Rwanda’s female representation in positions of influence is well known [13]. For example, the constitution requires that at least 30% of elected senators in parliament are women [21], and as a result 56% of lawmakers were female in 2010 [22]. Women have the right to inherit assets according to the 1999 law of succession [23]. It is evident that women parliamentarians have been empowered both legislatively and politically, and such empowerment can influence SRHR policies. There is a body of research that has looked at the impact of women’s representation in parliament as distinct from their knowledge, attitudes, and perceptions of SRHR. However, scholars disagree on the impact of women in politics. While one assertion is that female parliamentarians have different resources and interests in gender to their male counterparts, meaning they can achieve different outcomes [24, 25], others think the increase of female representation in parliament only changes the social climate within the political arena to guarantee a gendered agenda, but has little impact on policy outputs [25, 26]. The purpose of this study was to explore the empowered perspectives of women leaders in Rwanda about the recent policy change for safe abortion given their purported influence of other women and female adolescents. The study identifies women leaders’ perceived barriers and their attitudes about resulting consequences toward safe abortion, which helps provide insights into their alignment with the new law as well as their willingness to support and advocate for it.

## Methods

### Study design

A qualitative study design used to explore women leaders’ perceptions of safe abortion as provided for under Rwandan law. Data were collected through focus group discussions (FGDs) and key informant interviews (KIIs) conducted with female representatives who are part of the local community. The data were subsequently analysed using qualitative content analysis, a method suitable for exploring manifest and latent content and meanings expressed by participants [27].

### Setting and participants

Three districts were purposively selected to recruit participants. All participants are from Gasabo, Kicukiro, and Nyarugenge districts. The settings were chosen because of their greater incidence of abortion [28].

Participants were women who occupy leadership positions either in government or civil society organizations (CSOs). In total, 51 women participated in seven FGDs, each consisting of five to seven participants, and eight KIIs. Women leaders at different levels – from parliamentarians to village-level representatives – were represented in all FGDs and KIIs.

The sociodemographic characteristics of the participants are presented by district in Table 1. Of the 51 participants, 15 were educated to at least a university level, 20 to a secondary school level, and others achieved either a primary school level of education or vocational training. The majority of participants were skilled and employed professionals working in parliament or civil society or as CHWs in various villages of the selected districts. The age of the participants ranged from 28 to 60 years.

**Table 1:** Sociodemographic characteristics of the study participants

District	Site	# of FGD (N = 7) # of KII (N = 8)	# of participants (N = 51)	Average age (range)	Marital status	Occupation	Education level
Gasabo	Berwa	FGD 1	5	44 (39–55)	Married (3) Widowed (2)	Tailor/social affairs (2) Farmer/social affairs (3)	Vocational (2) Secondary (2) University (1)
	Ururembo	FGD 2	7	39 (31–49)	Married (7)	Tailor/CHW (2) CHW (5)	Primary (3) Secondary (4)
	Rugazi	FGD 3	6	38 (28–50)	Married (5) Widowed (1)	Farmer/CHW (1) CHW (4) Tailor/CHW (1)	Primary (1) Secondary (5)
Kicukiro	Niboye	FGD 4	6	52 (43–59)	Married (5) Divorced (1)	CHW (6)	Primary ( 3) Secondary (2) University (1)
Nyarugenge	Nyakabanda	FGD 5	6	42 (30– 56)	Married (5) Single (1)	CHW (6)	Primary (4) Secondary (2)
	Munini	FGD 6	6	38 (29–41)	Married (4) Single (2)	CHW (6)	Primary (1) Secondary (5)
Kigali	NWC	FGD 7	6	41 (36–45)	Married (3) Single (1) Widowed (2)	WMO (2) WEO (2) ERMO (2)	University (6)
Kigali	Govt and CSOs	KIIs	8	47 (36–60)	Married (5) Single (2) Widowed (1)	SA (1) MP (3) GMO (1) ED (1) PO (1) ES (1)	University (all)

ED: executive director, ERMO: entrepreneurship and rural mobilization officer, ES: executive secretary, GMO: Gender Monitoring Office, MP: member of parliament, NWC: National Women’s Council, PO: program officer, SA: state attorney, WEO: women empowerment officer, WMO: women mobilization officer

### Interview guide

The research team developed an interview guide based on the existing abortion law in Rwanda [19, 20], and the available scientific literature on safe and unsafe abortion [28–33]. The questions were: “What is your opinion regarding provision of safe abortion as provided for by the abortion law in Rwanda?”, “What are the consequences experienced by young women seeking safe abortion?”, and “What are the barriers for seeking safe abortion?”. Before the first FGD, a pilot test of the interview guide was carried out with women in the outskirts of Kigali City to ensure that questions were clear and understandable. The women who participated in the interview test did not participant in the actual data collection. After this process, minor adjustments were made to the interview guide.

### Data collection procedure

Women in leadership positions were contacted and asked to participate in the study. They were interviewed from a quiet place of their choice. Some chose to be interviewed at their place of work, others at cell offices, others under the tree shade, while others chose hotels premises. Eligible participants, who fulfilled inclusion criteria and were willing to participate, were informed in detail about the study objectives before signing the consent form.

We did a mapping of organizations or political positions where women leaders are found such as parliament, NGOs, Civil society organizations and in the decentralized government. From this mapped list we interviewed those leaders that accepted to be interviewed.

Among those that accepted to be interviewed, such as members of parliament and leaders of none- governmental organizations could only be interviewed by use of KIIs due to their different schedules and researchers could not get many at the same time too form a FGDs.

There were however, other targeted participants that were easy to form FGDs such as community health workers, social well affairs at cell/village level because researchers could easily find more than two in the same village or combine villages to form FGDs.

### Ethics consideration

Permission to conduct this study was authorized by the institutional review board of College of Medicine and Health Sciences under approval Number: No 347/CMHS IRB/2019.

All KIIs and FGDs were conducted in October 2019. Interviews were performed by a moderator (LR) and a co-moderator (AK). A note-taker/observer was also present at all FGDs, which were performed in Kinyarwanda, the local language. Probing questions were asked to further explore the opinions of the participants. The discussions were digitally recorded and interviews lasted on average 60 min.

After six FGDs, no major new information was being brought up in the discussions. To substantiate the saturation of data, a seventh FGD in a different district was performed, as well as a seventh KII. The recordings were transcribed into Kinyarwanda and later translated into English. To ensure the accuracy of the translations, parts of the translated transcripts were back-translated into Kinyarwanda and show no important discrepancies.

### Analysis

An inductive qualitative content analysis was applied to analyse the transcribed materials and formulate the themes, categories, and sub-categories, to explore the manifest and latent content of the interviews [27]. First, the text was read through several times to fully understand the content and identify areas of interest as per the study objective. Meaningful units of content addressing the aim of the study were identified, then condensed and labelled with codes. These codes were compared for similarities and differences resulting to sub-categories. The sub-categories were then compared to form categories. LR and AK shared responsibility during these stages of analysis. A theme emerged during the analysis illustrating the latent content of the data [27]. All data were reviewed several times to ensure that no relevant material was left out. To boost the trustworthiness of the findings [34], results were discussed and reflected on by all members of the research team.

## Findings

The emerging theme of the women leaders’ perceptions is: *Strong barriers and numerous consequences of safe abortion*. Two categories developed from this theme are: (1) *Perceived barriers of safe abortion* and (2) *Perceived consequences of safe abortion.* The first category is divided into the following sub-categories: *Reluctance to fully support safe abortion due to perceived unjustified abortions*, *Abortion is against our culture Abortion is against our religious beliefs*, *Abortion-related stigma, “Emotional attachment to the unborn* and *Lack of awareness of abortion law*. The second category is divided into the following sub-categories: *Physiological trauma*, *Cause of barrenness/infertility*, *Increase in abortion services abuse by adolescents/women*, *Increase of workload for healthcare providers (HCPs)*, and *Increase in sexual activities and STIs*.

Table 2 presents an overview of the theme, categories, and sub-categories that emerged from the analysis. This section will provide a summary of the category, followed by a presentation of its sub-categories. Quotes that illustrate participant views from FGDs and KIIs are presented in italics.

**Table 2:** An overview of theme, categories, and sub-categories

Theme	Categories	Sub-categories
Strong barriers and numerous consequences of safe abortion	Perceived barriers of safe abortion	Reluctance to fully support safe abortions
		Abortion is against our culture
		Abortion is against our religious beliefs
		Abortion-related stigma
		Emotional attachment to the unborn
		Lack of awareness of abortion law
	Perceived consequences of safe abortion and reluctance to support the removal of the court order	Physiological trauma
		Cause for barrenness/infertility
		Increase in abortion services abuse by adolescents/women
		Increase of workload for HCPs
		Increase in sexual activities and STIs

### Perceived barriers to safe abortion

Although women leaders are aware of the large number of abortion-related deaths among adolescents, some still feel that providing safe abortion services is tantamount to murder. They described instances where safe abortion is perceived as unnecessary. For example, a situation where a woman commits adultery and gets pregnant while her husband is serving a long prison sentence, and she is worried about potential repercussions on the husband’s release. In this case, some participants consider that the woman should have no right to access safe abortion services.

### Reluctance to fully support safe abortion due to perceived unjustified abortions

Participants criticized current national efforts as focusing only on the provision of safe abortion rather than on raising awareness and preventing unwanted pregnancies. They would like to see more efforts directed toward preventing unwanted pregnancies among adolescents instead of toward discussion and provision of safe abortion. They suggest that all stakeholders need to be brought on board to ensure effective preventive measures, and mentioned inclusion of parents, teenagers, health providers, and religious leaders to encourage a common understanding toward prevention.

Additionally, when female MPs were asked if they would freely talk about safe abortion in their community, almost all of them indicated that regardless of age, or experience of rape or incest, abortion is never justified. But they do accept that safe abortion may be warranted for health reasons.

*“There is a bad connotation on hearing ‘abortion’ in Kinyarwanda. The word ‘abortion’ disturbs. Even with safe abortion, the word ‘abortion’ outweighs the word ‘safe”* (MP 1 KI1I).

*“Why are all efforts toward killing instead of educating the people? I think all efforts should be directed toward awareness for prevention, and we can never fail because we have succeeded with bigger tasks in this country”* (Participant 2, FGD 7).

Participants were asked from a body autonomy perspective if they think that women who get pregnant as a result of rape have any rights over their own bodies. One replied:

*“Yes, she has rights, if raped… but should not abort because the unborn baby has a right to live too. Rights should not be absolute at the expense of others”* (MP 3 KII 3).

In one FGD, the six participants were asked about circumstances where a teenager is too young to give birth. They were asked if they would regard this murder, or instead if it is saving the mother’s life. One replied, *“It is killing because the baby is already alive. Actually what we are discussing is killing.”* (Participant 2, FGD 1).

*“When an adolescent is made pregnant by her father or brother, it’s a big problem. But even then, they should give birth to the baby regardless of how they will take care of the baby.”* (Participant 4, FGD5).

### Abortion is against our culture

Even though women leaders acknowledge that many adolescents die as a result of unsafe abortions, they think that those adolescents should be taught that once a pregnancy happens, a child has to be born. They feel they cannot promote safe abortion because of their cultural beliefs.

The reluctance of these leaders to support safe abortion is partly due to their fear that Rwandans are not willing to listen to any messages on the topic due to the subject’s sensitivity.

*“I don’t know if Rwanda’s culture will embrace the safe abortion. You need research to know how Rwandans will receive this message. You know laws are made for Rwandans but you should pay attention to how they interpret them […] it will not be well received”* (MP 2 KII 2).

Participants raised the concern that, in Rwanda, when you are talking to people it should be to those who are listening and are able to understand the message. They gave the example that when leaders talk about development, the masses pay attention. But when the subject of abortion is discussed among Rwandans, deep consideration must be given to how it will be interpreted.

According to participants, people find it very difficult to confront the issue; they think that rather than focusing on safe abortion, more efforts should be geared toward prevention and abstinence. They advocated for safe abortion on medical grounds, and in situations where it is unlikely the fetus will survive.

*“We told you the truth as parents; it is difficult to tell a parent that if your child has an unwanted pregnancy, this [abortion] is how to help her”* (Participant 3, FGD 7).

*“In our culture, aborting is a sin. Our culture does not accept it; it’s a bigger challenge for people to encourage what they don’t believe in”* (MP 2 KII 2).

One of the study participants was not very enthusiastic to encourage safe abortion for those who need it, asked why more effort is not put into preventing unwanted pregnancy before resorting to provision of safe abortion.

Another participant in one of the FGDs composed of social affairs workers at the cell level questioned who would be culpable if she encouraged an adolescent to abort. She feels she would be culpable herself.

*“Myself, as a woman and as a former care provider, you cannot approach me for assistance to abort, I can’t manage it. I can direct you to others, but I can’t manage it.”* (Participant 4, FGD 7).

*“For me, of all available solutions shouldn’t be to legalize ‘killing’. There are other solutions. Instead there should be more promotion of condom use to prevent pregnancy and STDs. To me this [abortion] is not the first solution to ponder about”* (Participant 3 FGD2).

A participant in an FGD comprising CHWs from Kigali is also reluctant to support safe abortion, saying:

*“Let me tell you, those things are difficult to say – that there’s a service at the health facility, go there tomorrow morning … and abort if you don’t want to give birth to that child. I can’t manage”* (Participant 2, FGD 2).

### Abortion is against our religious beliefs

Some of the women leaders from the 7th FGD were firm in their belief, to the extent of suggesting they would resign from their positions if they were required to provide information about safe abortion to their communities.

*“When it’s an embryo it’s already a human being in the eyes of God, so aborting it is killing a person. Those who believe in God cannot promote such a practice”* (MP 2 KII 2).

*“Me, I cannot go there, grab a microphone and encourage fellow women to go and kill those in their womb. Anybody who knows the joy of having a child cannot do it […] maybe men can because they don’t become pregnant”* (Participant 5, FGD 7).

Women leaders reiterated that mothers have rights over their bodies but so does the unborn fetus.

*“Mothers have rights but also the unborn fetus has rights. You cannot put your own rights above the rights of the unborn. The child already exists, period. This is how I understand it. I can’t really support safe abortion”* (Participant 2, FGD 6).

### Abortion - related stigma

It was the view of participants that abortion-related stigma influenced women’s immediate course of action when confronted with unwanted pregnancy instead of the existing abortion laws. Skeptics of safe abortion cited culture and religious issues that further hinder their seeking of safe abortion. This was evidenced by one participant, quote:

*“Those who have aborted are not accepted in the community. Women choose to move to other localities just to cover up or cleanse their reputation”* (Respondent 3, FGD 2,)

### Emotional attachment to the unborn

Alongside identifying faith-related barriers, some leaders describe themselves as mothers with emotions that are different from men’s emotions. They feel that a mother’s merciful feelings toward children will not allow them to accept abortion, that it is a violation of the rights of the unborn child. They attribute their reluctance to encourage abortion to having been raised to believe that any child should be born regardless of the circumstances.

*“Women are always merciful…, instead of thinking about safe abortion; a merciful mother will say ‘when this baby is born we will collectively raise it. I don’t have a grandchild and I cannot let this baby be killed […] An embryo is already a child”* (Participant 6, FGD 6).

Participants went on to illustrate their difference from men by giving the example of ‘mad women on the street’ and how they handle their babies – suggesting that for women, no matter what condition they are in, children take priority irrespective of the available resources. They considered a situation where a grandparent raises a grandchild who then has an unwanted pregnancy. They say that, even then, grandparents cannot support abortion.

*“I raised you after your mother gave birth to you and gave you to me, give birth to that baby and I will raise it as long as I live. And if not, you can raise the baby too*” (Participant 4, FGD 4).

*“The reason men may support safe abortion is because it’s men who cheat on their wives. They say whoever tells me I got her pregnant, I’ll tell her to abort […]women are visionary, they’re preventing adultery, men are going to benefit”* (Participant 6 FGD 3).

#### Lack of awareness of abortion law

In order leaders to raise awareness about the law, leaders themselves should have reasonable knowledge about the law. It was from this perspective; the knowledge of some women leaders about the abortion law was sought from community health workers (CHWs) and social affairs workers as key influencers of women in the community.

They were asked to share their knowledge in relation to “the ministerial order N°002/MoH/2019”- which scrapped the requirement to go to court in order to seek an abortion. They were also to name any grounds on which one can seek safe abortion (i.e. the five grounds for seeking safe abortion).

Apparently and CHWs and social affairs workers indicated they did not know about them: except CHWs indicated they knew abortion could be sought on medical grounds to save the mother’s life.

The knowledge of CHWs and social welfare staff was illustrated in the following quotations.

*I know nothing about ministerial order of 2019 […] what is it? …what is it intended for?”* (Participant 2 FGD 6).

When asked under what circumstances one can seek safe abortion, majority of in a FGD composed of majority social well affairs said, “*We don’t know”*(Participant 6 FGD 6).

### Perceived consequences of safe abortion

While the ministerial order scrapping the requirement for a court order was seen by some as progress in the right direction, others viewed it with grave concern. Women leaders raised concerns and expressed fears that it may backfire. They suggested that abortion services might be abused because previously the court would investigate the circumstances of the pregnancy, but now people might see abortion as a quick fix for unwanted pregnancies.

### Physiological trauma

Women leaders also identified post-abortion trauma as a consequence of safe abortion. Participants expressed concern about the potential for trauma when the only option available is abortion.

Some participants advocated for promoting abstinence over safe abortion, and suggested that instead, severe punishments should be put in place for those who perform unsafe abortions.

*“A colleague of mine … who aborted is still traumatized to this day. It has haunted her; even after getting married and giving birth to children she continues to pray but cannot forgive herself”* (MP KII).

Participants expressed respect for women’s rights but at the same time they were firm in their unwillingness to support abortion. They reiterated that this applies even in the case of pregnancies arising from rape, citing the example of victims of rape during the genocide who gave birth.

*“Genocide victims of rape bore children and were traumatized, but abortion traumatizes even more and I think the baby should be born. Who knows what the future holds for the unborn?”* (MP2 KII 2).

### Cause of barrenness/infertility

Some participants cited the possibility of infertility as a consequence of safe abortion. They were concerned that even though it is considered safe, they doubt it is 100% safe.

“*There are those that abort and are not able to conceive again”* (Participant 5, FGD 1).

### Increase in abuse of service by women and adolescents

Participants were concerned that abortion services could be misused. They questioned what proof could doctor without base on without a court’s endorsement. They asked what documents a woman could take to the health facilities to show that she was impregnated by a brother, other relatives, raped or victim of other forms of sexual violence.

*“Services will be misused, at least court was a means to scrutinize all circumstances, such as genuine or none genuine rape”* (MP 1 KII 1).

*“Now men are going to impregnate women/adolescents with ease –a person laughingly told me that because safe abortion services will be accessed with ease”* (Participant 5, FGD 7).

### Increase of workload for HCPs

Participants anticipate an increase in workload for healthcare providers because the number of women seeking services will increase.

*“Truthfully, I don’t know how they will handle it – the service is complicated and will need a lot of employees. There’ll be so many women seeking the health services I don’t think health care providers will ever get time to rest”* (Participant 4 FGD 5).

After seeing that participants were concerned about changes to the abortion law, the interviewer wanted to know why, and asked:

Interviewer: So, do you find it a problem – making it simple to access safe abortion services?

*“Personally I don’t agree that making the law simple played a positive role. Accepting it remains a challenge for me*” (MP 1 KII 1).

Interviewer: “What can be done to remove those constraints?”

Participant: *“In order for people to not be afraid, there should be ongoing awareness. But I’m not going to endorse it, and I can’t steer anyone that might seek it from me”* (MP 3 KII 3*)*.

### Increase in sexual activity and STIs

Participants were also concerned that making safe abortion accessible without a court order will likely increase sexual promiscuity amongst adolescents, because abortion will be made easy. They predict it will be abused by some women in unstable relationships; for example, a woman might tell her husband or partner that she will abort his ‘useless kid’ and never see him again.

Other concerns raised were related to an increased risk of STIs. Some women leaders believe encouraging safe abortion would result in increased sexual activity amongst adolescents, thus increasing the incidence of HIV/AIDS transmission. They reiterated that adolescents are afraid of unprotected sex due to the risk of pregnancy rather than contracting HIV.

*“Adolescents used to be afraid of unprotected sex due to the shame of unwanted pregnancy. When abortion services are freely accessible, there’ll be more sexual activity and an increase in HIV/AIDS”* (Participant 7 FGD 2).

*“In most cases girls use condoms for preventing pregnancy rather than avoiding HIV infection. They say there’s palliative care for HIV – if safe abortion is freely provided, condoms will be ignored completely and HIV will increase”* (Participant 5, FGD 4).

## Discussion

This is the first study in the Rwandan setting exploring women leaders’ perceptions toward safe abortion. The emerging theme *Strong barriers and numerous consequences of safe abortion*” reflects how women leaders perceive safe abortion and its related consequences. The perceived barriers women leaders identified include *Reluctance to fully support safe abortions*, *Abortion-related stigma, Abortion is against our culture, Abortion is against our religious beliefs*, *perceived emotional attachment to the unborn and Lack of awareness of abortion law.* The perceived consequences identified are *physiological trauma*, *cause for barrenness/infertility*, increase in misuse of services by adolescents/women, *increase of workload for HCPs*, and *increase in sexual activities and STIs.*

It is evident from our findings that, where abortion is concerned, women leaders in Rwanda contend with similar provocations associated with the power dynamics and power relations as those found across Sub-Saharan Africa. Mistrust between genders, as well as with healthcare providers, in relation to abortion, is supported by a context of male superiority, which denies the subject of abortion for public, political, and medical professional discourse, and ignites the morality attitudes against safe abortion [35, 36]. This reality is critical and important component of programmatic intervention for safe abortion in low resource setting Sub- Saharan This antagonistic context is critical to understand as problematic among some women leaders in relation to the promotion of programmatic intervention for safe abortion across Sub- Saharan Africa [36]. Effective strategies aimed at increasing women’s access to safe abortion services, as well as working to shift the power dynamics, are both promoted and required to achieve better public health outcomes. However, and as our findings strongly suggest for Rwanda, the attitudes and perspectives of women leaders are yet to become aligned with these initiatives. Yet, understanding their voices takes an important first step towards acknowledging powerful and politically impactful barriers to safe abortion services for all childbearing-aged females in the country.

Previous studies conducted in sub-Saharan African countries agree with this study. A Ghanaian study using semi-structured in-depth interviews among adolescents (aged 13–19 years) who had been pregnant at least once, indicated barriers similar to those reported in this Rwandan study. These include poor access to safe abortion services, poor awareness of the safe abortion law, stigma, high costs, negative consequences such as death and hemorrhage, barrenness, and lack of confidence in care professionals [31].

This study shows that abortion-related stigma, feeling guilty to culture and religion norms were some of the consequences of abortion. Likewise a qualitative study that explored the experiences of women in Kenya regarding their decision-making process prior to seeking an abortion shows that women went for an unsafe abortion due to a lack of social support or support from their male partner, perceived deviance from family or societal expectations, and a culture of pregnancy out of wedlock [37]. The reluctance of women leaders to support safe abortion due to religious, moral, and pressures of cultural convictions was very pronounced during this research. This is similar to findings from research conducted in Ghana that assessed provider-related obstacles and post-abortion care. Both studies suggest, providers were more driven by religious values, hence considering abortion sinful. Their faith and morals about the sanctity of the foetus contradicted their duty to provide safe-abortion care. In addition, social pressures (opinions of peers concerning abortion) affected providers’ decision to openly provide abortion services [38, 39].

In this study, the views of those against safe abortion were expressed more prominently than those who support safe abortion. Participants’ skepticism to support safe abortion is related to various issues, from concern that safe abortion services will be abused, to an increase in STIs, to potential infidelity and trauma. Perceived stigma and abortion-related trauma was also reported in other studies conducted in Ghana, one involving a weighted sample of 1,880 women aged 15–49 [31, 40].

However, whereas in this study the women leaders vehemently discourage safe abortion on all grounds except medical, the Ghanaian study reveals that parents and guardians encourage adolescents to abort [31]. The main influencers in the decision-making process when adolescents became pregnant were parents, friends, or family members. Parents were found threaten to disown their child if they did not abort, while other adolescents chose to leave their homes to live with other families in order to evade pressure from their parents or family to abort [31].

In this study, participants did not make any distinction between safe and unsafe abortion – they perceived all types of abortion to have the same consequences. Yet a qualitative study conducted in Ghana and published in 2019 illustrates that most men know the difference between safe abortion and unsafe abortion, and would support their partner to have a safe abortion [3]. Furthermore, it is important to add that while this study used qualitative techniques, most of the other studies discussed here were conducted using quantitative techniques. The differences between the Rwandan study and these other studies can probably be accounted for by differences in cultural openness and awareness of the law.

### Methodological considerations

The major strengths of this study are the purposive selection of study participants from both government and CSOs, with varying educational levels. These women leaders are directly or indirectly involved in efforts of empowering women, and so their opinions and views have gravitas. The FGDs included women who willingly discussed all aspects of SRH, with a special focus on abortion. The data collection tool was presented to a wider research audience for input and validation. The research team collaborated throughout the entire process until this article was finalized. As a practice in qualitative research, credibility, dependability, transferability, and confirmability of the findings were taken into account to ensure trustworthiness [34, 41]. During the FGDs, a comfortable environment was created to enable free contribution from all participants and avoid power asymmetry between interviewee and interviewer, which can potentially negatively impact the research findings [42].

However, it should be noted that participants are members of Rwandan society; the prevailing norms, customs, and taboos surrounding abortion do influence their responses. Secondly, Rwanda is patriarchal society with different gender views about abortion. However, the research team consisted of a medical doctor with experience in public health research and a public health professional with a specific focus on social research. With the attention given to trustworthiness, we do believe our findings reflect the experiences of other women leaders in the country.

## Conclusions

The subject of safe abortion evoked mixed and emotional reactions amongst participants, and in most cases the words ‘safe’ and ‘unsafe’ were used interchangeably. The study found that the word ‘abortion’ disturbs – even when talking about safe abortion, the word ‘abortion’ overshadows the word ‘safe’. Participants’ skepticism toward safe abortion was mostly premised on the potential abuse of abortion services due to the abolition of the court order, and their religious beliefs and cultural expectations.

CHWs and social affairs workers had little or zero knowledge of the grounds on which one would seek a safe abortion, and no awareness at all of the removal of the court order before seeking a safe abortion. There is a need for a paradigm shift in the current mindset of women leaders in relation to safe abortion. This can be achieved through using mass media to raise awareness about legal safe abortion to the general population. Integrating safe abortion services into health centers and giving CSOs a role to play will be essential. Furthermore, using fora of women leaders gathering and using political leaders will be a good option targeting women leaders.

## Data Availability

The person to be contacted if someone wants more information about the data of this study is Dr. Rugema Lawrence who is also the corresponding author of this manuscript. However, due to confidentiality it remains anonymous and unavailable to the public. It is only accessible by the research team currently.
